# Teaching content in practice: Investigating rehearsals of social studies discussions^☆^

**DOI:** 10.1016/j.tate.2019.06.017

**Published:** 2019-11

**Authors:** Sarah Schneider Kavanagh, Chauncey Monte-Sano, Abby Reisman, Brad Fogo, Sarah McGrew, Peter Cipparone

**Affiliations:** a University of Pennsylvania, 3700 Walnut St., Philadelphia, PA 19104, USA; b University of Michigan, USA; c San Francisco State University, USA; d Stanford University, USA

## Abstract

Despite evidence of its benefits, discussion remains rare in history/social science classrooms. To address this problem, communities of teacher educators (TEs) have begun supporting novices to approximate discussion facilitation. Some scholars are concerned that this turn to practice will come at the cost of content preparation. Focusing specifically on rehearsals of discussion facilitation in three history/social science methods courses, our analysis investigates whether, how, and in what ways TEs worked on content while engaging novice teachers in practicing discussion facilitation. We found that TEs found ways to work simultaneously on content and practice during rehearsals of discussion facilitation.

## Highlights

• Debates exist about whether focusing on practice in teacher education requires reduced focus on content.
• It is possible to work on developing novice teachers' content knowledge and practical skill simultaneously.
• Rehearsals can focus on supporting students to attend to the structure of the discipline.
• Rehearsals can focus on supporting students to construct and verify knowledge in disciplinarily appropriate ways.
• Rehearsals can focus on making content accessible and anticipating how students will think about content.

Despite widespread agreement about the value of student-to-student talk in classrooms (Hess, 2009; Hess & McAvoy, 2015; Hess & Posselt, 2002; Kavanagh & Rainey, 2017; Parker et al., 2011; Reisman et al., 2018), productive classroom discussion remains rare (Cazden, 2001; Nystrand, Wu, Gamoran, Zeiser, & Long, 2003). This is particularly true in history and social science classrooms (Bain, 2006). By contrast, the frequency of lecture and individual textbook work begs the question: why have instructional methods been so slow to change? One answer to this question is what Kennedy (1999) calls “the problem of enactment,” or the gap between what teachers can envision and what they can enact. From this perspective, the development of professional consensus about the value of discussion cannot shift what happens in classrooms without a parallel development of a teaching force that can act on that consensus.

Developing a teaching force that regularly engages students in productive discussions will require a concerted effort in teacher education. How best to support novice teachers to facilitate high quality discussions about history and social science is not a simple question. However, it is one that researchers have begun to investigate. In their study of how to prepare professionals for complex practice, Grossman, Compton, et al. (2009) and Grossman, Hammerness, and McDonald (2009) offer one answer: a framework that names *representations* of practice, *decompositions* of practice, and *approximations* of practice as central pedagogical approaches to preparing professionals. Following the logic of the Grossman et al.'s (2009) framework suggests that preparing novice history/social science teachers as discussion facilitators will require showing novices examples of discussions, breaking the practice into digestible components, and engaging novice teachers in trying out discussion facilitation in environments where stakes are low and supports are plenty.

Many teacher educators (TEs) have used Grossman et al.'s framework to inform pedagogical and curricular changes to their work with novice teachers (Ball, Sleep, Boerst, & Bass, 2009; Ghousseini & Herbst, 2016; Kavanagh & Danielson, 2019; Ross & Cartier, 2015; Von Esch & Kavanagh, 2018). These changes are representative of a larger shift in teacher education, what Zeichner (2012) has called “the turn to practice.” The turn to practice is a widening of focus among scholars of teacher education from an emphasis on identifying necessary knowledge for teaching to a focus on supporting novices to develop and use knowledge in action (McDonald, Kazemi, & Kavanagh, 2013). While the practice turn has taken a firm hold in some teacher education communities, it has not been without controversy (Philip et al., 2018). Time is a finite resource in teacher education and some scholars have raised questions about whether increasing the amount of time spent learning in, through, and about practice will necessarily take time away from other vital aspects of teacher education. One such vital aspect of teacher education is novices' development of content knowledge. Will a broadening of focus to work on both knowledge *and* action in teacher education require that less time and attention be paid to the development of specialized content knowledge (Ball, Thames, & Phelps, 2008)?

As scholars whose work rests on an assumption that the sophistication of a teacher's practice is in a direct relationship with that teacher's content knowledge (among other things), we investigated the relationship between practice-based pedagogies of teacher education and the *content* preparation of teachers. To investigate this relationship, we conducted a comparative case study of three history/social science methods courses at three institutions. This article is the product of one line of inquiry embedded in a larger investigation. In other articles, we present findings pertaining to the role of content knowledge in how TEs decomposed and represented the practice of discussion (Kavanagh & Rainey, 2017; Reisman et al., 2018; 2019). The findings we report in this article, however, arose out of a targeted analysis of all data pertaining to how TEs engaged teachers in *approximations* of discussion facilitation; specifically, we examine rehearsals of discussion facilitation in history/social science methods courses. We grounded this targeted analysis in the following questions:

• How do history/social science methods instructors who explicitly focus on teaching discussion facilitation guide novice teachers in approximating discussion facilitation during rehearsals?
• To what extent and in what ways do history and social science TEs work on *content* in approximations of discussion facilitation during rehearsals?

## 1 Content knowledge for history/social science teaching

Content knowledge includes information about specific topics, as well disciplinary practices and concepts. In U.S. schools, the social studies primarily include four disciplines – civics, economics, geography, and history – so those disciplines tend to dominate the topical knowledge, disciplinary practices, and disciplinary concepts emphasized in U.S. schools (e.g., NCSS, 2013). Schools, standards, and high-stakes assessments typically emphasize information about specific historical topics as the core content for students to learn (e.g., Bain, 2006; Cuban, 2016; Ravitch & Finn, 1987). While topical information is important, it is only one aspect of content knowledge (e.g., Wineburg & Schneider, 2009/10). We include the disciplinary work of experts—both the practices they engage in and the concepts that guide them—as aspects of content knowledge. Much as Schwab (1978) highlighted the procedural and substantive knowledge that form the structure of a discipline and the process of constructing knowledge in a discipline, we attend to what disciplinary experts do and how they think as aspects of content knowledge. For example, disciplinary practices in history can include analysis of sources (e.g., sourcing, contextualizing, and corroborating—Wineburg, 1991), developing interpretations on the basis of evidence (e.g., Monte-Sano, 2010), and considering alternative or challenging perspectives (e.g., Collingwood, 1943). Disciplinary concepts in history can include understanding evidence, interpretation, causation, change, context, time, and other concepts that orient one to the study of the past (e.g., Lee, 2005). Together, these practices and concepts make up historical thinking and understanding, key aspects of social studies content knowledge that enable students to understand the past and how interpretations are constructed, construct their own interpretations, and critique the interpretations of others.

To cultivate students' content knowledge, teachers must understand the content themselves, as well as how to convey that content to students. Shulman (1987) identified pedagogical content knowledge (PCK) as an “amalgam of content and pedagogy that is uniquely the province of teachers” (p.8). PCK includes knowledge of how students make sense of content as well as how to represent content to students so that they understand it. PCK relies on teachers' subject matter knowledge, which Ball et al. (2008) further specified to include “specialized content knowledge,” the knowledge teachers need to be able to teach content to others that is not often needed outside of teaching (p. 400). For example, when teaching a historical issue that requires interpretation, teachers might need to understand the range of historical interpretations possible and which pieces of evidence support each interpretation, rather than settling on one interpretation (e.g., Monte-Sano & Budano, 2013). Thus, content knowledge is both a goal for student learning as well as a requirement for teachers to achieve this goal. To achieve this goal involves more than just knowing a lot of information, but also the structure of the discipline, disciplinary practices and concepts, and an understanding of how to work with students on content.

### 1.1 Core practices of teaching

Reform efforts in teacher education's recent history have focused primarily on the structural elements of preparing teachers (Grossman, Kavanagh, & Dean, 2018). Such structural elements include identifying the knowledge necessary for teaching, extending teacher education into graduate education, creating professional development schools (The Holmes Group), aligning renewal efforts in teacher education with renewal efforts in K-12 schools (John Goodlad's Network for Educational Renewal), highlighting the role of Arts & Science faculty in teacher education, extending teacher education into induction programs for early-career teachers, and developing data systems for teacher education programs to assess their impact on novice teacher and K-12 student learning (Teachers for a New Era). What reform efforts from the past 25 years have largely ignored are the *instructional* aspects of how teachers are prepared. Recent work on core practices of teaching and practice-based teacher education has been grounded in a belief that instructional practice, both of K-12 students and of novice teachers, is a particularly promising lever for improving student learning (Ball & Forzani, 2009; Grossman et al., 2009; Grossman, Kazemi, Kavanagh, Franke, & Dutro, 2019; Hauser & Kavanagh, 2019). Scholarship on core practices and practice-based teacher education is based on the idea that “practice in complex domains involves the orchestration of understanding, skill, relationship, and identity to accomplish particular activities with other in specific environments” (Grossman et al., 2009). A core practice, therefore, is not a decontextualized behavior or static technique. Instead, it is the act of negotiating relationships and one's own identities and understandings while engaged in professional activity.

#### 1.1.1 Focal core practice: facilitating discussion

**Fig. 1:**
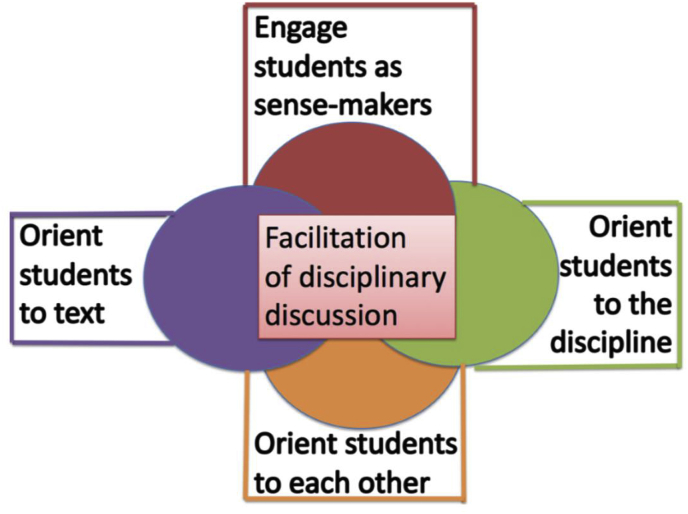
Framework for facilitating disciplinary discussion in history.

In this paper, we focus on the core practice of facilitating discussion and the opportunities that TEs have to focus on content while supporting novices as they develop as discussion facilitators. To develop a vivid picture of the practice of discussion facilitation, we first consider what counts as discussion. In defining discussion, we look to Parker and Hess (2001), who describe discussions as “text-based shared inquiry of the listening-and-talking kind” wherein *shared inquiry* refers both to the “dialogic relations involved in reading, writing, and talking” and to the fact that “inquirers hold in common the same object of inquiry, which is the discussion topic and material” (p. 275). We also draw on Kazemi and Cunard's (2016) and Kavanagh and Rainey's (2017) work on orienting moves, which was the bedrock of our previously published framework for the practice of facilitating historical discussions. Our framework involves four overarching practices: (a) engaging students as sense-makers; (b) orienting students to each other; (c) orienting students to texts as sources of historical knowledge and evidentiary warrants; and (d) orienting students to the interpretive practices of the discipline (see Fig. 1) (Kavanagh & Rainey, 2017; Reisman et al., 2018; 2019). The purpose of this framework is to specify the components of work involved in facilitating discussions in order to make elements of this core practice more visible for novice teachers and to support TEs' efforts to prepare novices to facilitate discussion.

For classroom discussion to occur, a teacher must engage students as sense-makers and provide them with tasks that require interpretation or creativity, rather than treat discussions as opportunities to quiz students with narrow questions about what they already know (Cazden, 2001). The second component of our framework focuses on orienting students to each other. Sociocultural theory holds that knowledge construction occurs through social interaction, which supports the development of “higher mental functions” (Leont'ev, 1981, pp. 55–56). Learners build new knowledge through social engagement that is mediated by cultural tools (and in this case, *disciplinary* tools), such as language, text, and shared practices. In order to support this kind of collective knowledge construction, teachers must help students grapple with each other's ideas. Instead of asking students to serially report out their ideas, teachers who orient students to each other build a community where ideas and knowledge are developed together. From this Vygotskian perspective, the teacher's facilitation and orchestration of student voices scaffolds the complex mental processes that students will ultimately internalize.

Sociocultural perspectives of learning also inform the third component of our framework—orienting students to the text. From a sociocultural perspective, tools shape social engagement and individual learning by carrying shared cultural and historical meaning across people and time (Cole & Wertsch, 1996). Texts serve as tools, or shared disciplinary artifacts, around which classroom discussions revolve. During discussion, teachers work to orient students to text at several levels. A teacher might ensure that students have a common focus. Teachers also orient students to text when they check that students comprehend texts at a literal level and have a foundation upon which to develop interpretations. At the most complex level, teachers orient students to text by framing texts as warrants and ensuring that claims are supported with textual evidence.

**Fig. 2:**
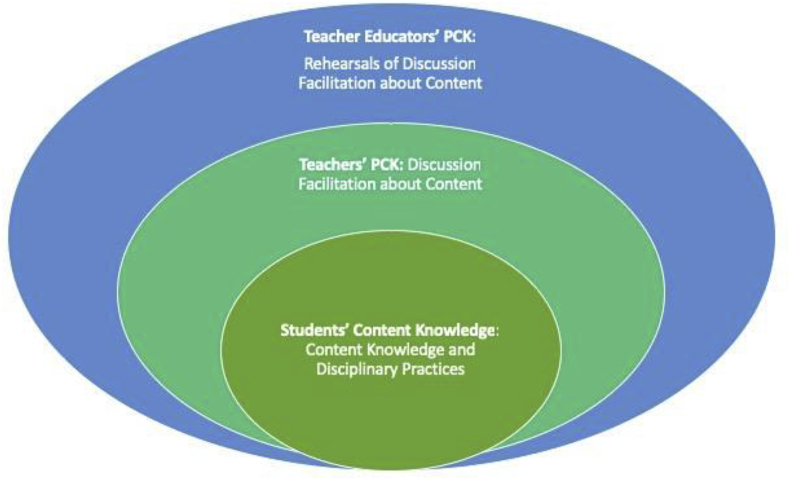
Model of teacher educators' PCK for discussion facilitation.

Finally, as Kavanagh and Rainey (2017) argue, in discussions students need to be oriented to the inquiry practices of one or more disciplines, in this case history and social sciences. Although orienting students to texts ensures that they grapple with content, disciplinary work—in history and social sciences—entails a process of inquiry-driven knowledge construction (Schwab, 1978). In the context of a text-based history or social science discussion, students who engage in knowledge construction should leave the discussion with a new or more complex understanding of the topic, as well as an understanding of *how that knowledge was constructed*. Teachers can orient students to the syntactic structure of the discipline (Schwab, 1978) by explicitly linking student contributions to the interpretive practices of the disciplines. At the substantive level, teachers can orient students to the disciplines by highlighting the conceptual structures and disciplinary knowledge that shape expert work. A teacher who explicitly connects points in a discussion to conceptual understandings about history, for example (e.g., by referencing continuity and change, evidence, perspective, empathy, and causation (Lee, 2005; Seixas & Morton, 2013) helps orient students to the discipline. Teachers might prompt students to evaluate multiple perspectives about an event as they consider and evaluate different sources of evidence about a controversial public issue (e.g., Dimension 3, NCSS, 2013). Or, teachers could challenge students to investigate the worldviews of people living in a past time period, building their capacity for historical empathy. In short, the framework for facilitating historical discussions captures the complexity of teachers' PCK, as they engage in sophisticated pedagogical maneuvers to develop students' subject matter knowledge (see Fig. 2).

### 1.2 Teacher education pedagogy

Discussion facilitation as we describe it above is a complex practice that takes effort to learn. But what type of activity can teachers engage in that will support their learning of a complex practice? At its heart, this is a question about the pedagogy of teacher education – the activity that TEs engage teachers in to support their learning of practice. Because teacher education research has focused more on issues of curriculum and structure than on issues of pedagogy, we know much more about *what* we teach prospective teachers (e.g., how to facilitate discussion), than about *how* we teach them those things (Grossman, 2005). In this section, we draw on the limited scholarship on teacher education pedagogy to unpack the pedagogical approaches that TEs use to engage novice teachers in learning to enact sophisticated practice. In particular, our investigation rests on Grossman et al.'s (2009) framework for teaching practice – a framework that presents three pedagogical approaches to preparing novices for professional practice. The first of these three approaches is providing novices with representations of professional practice to help them see certain facets of practice that might be invisible to a novice otherwise. The second approach is offering novices decompositions of professional practice that break down complex practices into their constituent parts. The third, and final, pedagogical approach is engaging novices in approximations of practice, which offer novices opportunities to engage in what Ericsson (2002) calls “deliberate practice,” or the trying-on of challenging components of practice in sheltered environments.

#### 1.2.1 Focal teacher education pedagogy: rehearsals

One way to support novices in approximating the practice of discussion facilitation is through rehearsal. As described by Lampert et al. (2013a, 2013b), rehearsals of discussion facilitation involve one novice teacher taking responsibility for facilitating a discussion in a simulated classroom while other novice teachers participate in the role of students. The final role in a rehearsal belongs to a TE who offers feedback and creates experiences within the rehearsal by participating in various ways (e.g., acting as a student to contribute ideas that students are likely to have; acting as a teacher to model something; pausing the action to offer feedback, asking a question or facilitating a short discussion). This structure for approximating practice is grounded in theories of professional learning. By frequently interjecting to highlight, offer feedback, or raise questions, the TE in a rehearsal is bringing conceptual elements to bear on particular problems. This weaving together of the practical and the conceptual is what allows novices in any profession to build *adaptive expertise,* or proficiency in both the central routines of a domain, but also the ability to make flexible judgments in the face of novel problems (Bransford, Derry, Berliner, & Hammerness, 2005). Skilled teachers need ways to make fast and flexible judgments during discussions using their own content knowledge and their knowledge of students, especially because the ways that students' content ideas emerge in a discussion are often unpredictable.

In facilitating rehearsals with novices around a core practice such as discussion, the skilled TE, then, also demonstrates PCK. In this case, however, the rehearsals serve as the pedagogical practice through which the TE develops novices' learning of *both* discussion facilitation and subject matter content (see Fig. 2). As such, this is the focus of our investigation: Within rehearsals of discussion, in what ways do TEs work on content?

## 2 Methods

The findings we report in this manuscript stem from an analysis of data across three history/social science methods courses at three universities. While our study spans three institutions, these three sites are only a portion of a much larger sample of twelve sites. The larger study in which this investigation is embedded is a qualitative, comparative, case study of twelve teacher education methods courses in different subject areas across eight U.S. institutions, all of which explicitly teach discussion facilitation. To investigate our research question, we draw on data from a two-year comparative case study (Yin, 2013) of teacher education pedagogy focused on teaching and learning core instructional practices for K-12 classrooms within history/social science methods courses at three institutions. Here, we focus on the second year of data related to rehearsals of discussion facilitation. We choose this year of data because it represented participating TEs' most recent efforts to run rehearsals after a year of learning through this research project.

### 2.1 Course contexts and participants

The methods courses under study were offered in the combined master's degree and certification programs at three different research universities during the 2015–2016 academic year. One TE from each of the three universities participated in this study: TE1-SEC, TE2-SEC, and TE3-ELE. TE1-SEC and TE2-SEC each taught secondary social studies methods courses that met for multiple semesters while novices held high school field placements. TE3-ELE taught the third course focused on elementary social studies methods and met for one semester while novices held K-5 field placements. These three TEs were a convenience sample from a group of social studies TEs working together as part of a cross-university research group, the Core Practice Consortium, led by Pam Grossman and Megan Franke. These TEs elected to participate in the study after discussing potential participants during a research group meeting. These participants worked in comparable programs that offered a master's degree and certification in one year, a similar orientation to content, and three or more years of teaching social studies methods while providing the contrast of elementary and secondary contexts. While all instructors are co-authors of this paper, the lead author was not one of the instructors.

The novice teachers in each methods course also participated in this study. They were invited to participate in the study by research assistants who were not the instructors of the study. All novice teachers consented to participate (n = 14 in TE1-SEC's class; n = 15 in TE2-SEC's class; n = 22 in TE3-ELE'class). Novice teachers' field placements within each program represented diverse contexts. In the elementary program, novices were placed in classrooms that ranged from socioeconomically, racially, ethnically, linguistically, and culturally diverse urban and suburban settings to homogenous, upper middle class urban and suburban settings. In the secondary programs, novices were placed in racially and economically diverse urban and suburban classrooms, though these also ranged from schools where students officially designated as English Language Learners represented the majority of students to competitive magnet schools where few students designated as English Language Learners were enrolled.

TEs and novice teachers primarily worked on the discipline of history in the secondary contexts, while civics, geography, and history were focal disciplines in the elementary context. Methods instruction in each course followed a pedagogical framework proposed by Grossman et al. (2009), in which novices were introduced to instructional practices through multiple *representations,* these practices were *decomposed* into their constituent parts, and novices were given multiple opportunities to *approximate* the practice in the context of methods instruction. Despite starting with a shared specification for whole-class discussion and a common framework for practice-based instruction, each TE drew on different technical terms and instructional activities to decompose the practice. We drew on Kazemi and Cunard's (2016) and Kavanagh and Rainey's (2017) work to develop the Framework for Facilitating Historical Discussions (see Fig. 1) *during* this year of methods instruction (after the elementary course was completed and while the secondary courses were in progress). The framework supported us to capture the patterns that we detected in novice discussion facilitation across elementary and secondary contexts.

#### 2.1.1 Elementary setting

TE3-ELE taught the elementary social studies methods course in the second semester of a 1-year certification and master's degree program. The course focused on core teaching practices that provide students opportunities to engage in social studies inquiry and related literacies. Prior to the discussion assignment, novices learned to elicit and respond to student thinking about time and change using historical photographs (as in Barton & Levstik, 1995) and to use strategy instruction to teach sourcing and contextualization (Wineburg, 1991). Discussion facilitation was presented as a distinct core practice essential to inquiry. Novices learned about facilitating whole-class discussion through experiencing and then preparing and rehearsing a visual inquiry lesson assignment (Johnson, 2013) before teaching it to their K-5 students and reflecting on their discussion facilitation. In a visual inquiry, students compare two images in light of a central question. This assignment is most similar to Parker and Hess's (2001) explanation of a Seminar in which consideration of a text's meaning is the main focus and developing an “enlarged understanding” of a text is the goal (p. 281).

Before rehearsals, the TE first modeled and represented the core practice by facilitating a discussion about two images of the Boston Massacre. Novices then decomposed the core practice both in that representation and in a video of an elementary social studies discussion. As novices developed their visual inquiry lesson, they were supported in refining the materials and lesson plan, identifying key details in their images that would yield meaningful inferences, and sequencing questions that moved students from observation (“What do you see?”) to inference (“What do you think?”) to interpretation or evaluation (“How does this help us answer our central question?” or “What does the artist want us to think?”). In this paper, we focus on the class session during which one novice rehearsed her full visual inquiry lesson with the whole class and then all novices rehearsed a portion of their lesson in small groups facilitated by the lead TE and assistants. For this paper, we focus on the rehearsals conducted with the lead TE who was the research participant. During rehearsals, the TEs paused novices to raise questions or offer feedback on how novices elicited student thinking, supported students in listening and responding to one another, or provided explanations to guide the discussion during the rehearsals with their peers. After rehearsals, novices taught their lesson to K-12 students and then submitted a video of their lesson, a lesson plan, and a reflection to the TE.

#### 2.1.2 Both secondary settings

TE1-SEC's secondary course included a two-semester sequence within a 1-year certification and master's program and TE2-SEC's course was part of a three-quarter sequence within a 1-year certification and master's program. In both courses, instruction on classroom discussion occurred during the winter. For both TE1-SEC and TE2-SEC, the fall courses introduced students to disciplinary historical literacy, in particular the skills of sourcing, contextualizing, corroborating, and close reading. In addition to developing their own curricular materials, students were encouraged to use and revise document-based lessons in the *Reading Like a Historian* curriculum (see http://sheg.stanford.edu/rlh). In TE1-SEC's and TE2-SEC's courses, novice teachers learned that the culminating whole-class discussion should focus on the lesson's central historical question, offering students an opportunity to bring often-contradictory documentary evidence to bear on the question. Novice teachers witnessed multiple representations of text-based whole-class discussion around a central historical question—through videos, classroom transcripts, and modeling by the TE.

In TE1-SEC's courses, novice teachers rehearsed discussion facilitation with their peers, first with materials from a lesson plan in the *Reading Like a Historian* curriculum, and then with materials that they developed for an assignment. To prepare for these rehearsals, novices used a planning scaffold that asked them to consider how each document responded to the central historical question, what issues of reliability were raised by each document, and what historical understanding they intended students to take away from the discussion. After rehearsals, novice teachers shared a 5 to 10-min video of their discussion facilitation with secondary school students, along with contextual information, relevant materials, and a reflection. Each novice also posted comments on three of their classmates' videos.

In TE2-SEC's courses, novice teachers worked with a partner to prepare sources and questions for use in a discussion that they practiced with peers during rehearsals. Several novices elected to use *Reading Like a Historian* curriculum. In this setting, all of the novices did not enact their rehearsed lesson with high school students in their placement, although novices did proceed to plan additional discussion lessons as part of a curriculum unit they constructed and enacted later in the year.

During rehearsals in both TE1-SEC's and TE2-SEC's secondary methods courses, the TEs both occasionally paused the facilitation to offer feedback or redirection, at which point the novice would proceed, attempting to incorporate the feedback. One difference was that TE1-SEC held whole-class rehearsals only, whereas TE2-SEC split the class in half and facilitated rehearsals with a small group while a teaching assistant did the same. We only focus on the small group rehearsals of the lead TE who was the research participant.

### 2.2 Data sources

We followed three history/social science TEs through two years of methods courses during which time participants discussed their practice with one another, watched video of each other's rehearsals, and refined their practice as rehearsal facilitators. From the larger data set, we sampled all data pertaining to TEs' facilitation of rehearsals of discussion facilitation during the second year of the study. Sampled data includes: course observation field notes, course artifacts (lesson plans, syllabi, power points, and assignments), video of rehearsals, video of participants discussing rehearsals with one another, and interview transcriptions. Interviews were conducted at the beginning and end of each participant's course and prior to every course session that included a rehearsal.

All together the lead TEs from the three sites led eleven rehearsals of discussion facilitation—TE2-SEC held two small-group rehearsals, TE1-SEC held four whole-class rehearsals, and TE3-ELE held one whole-class and four small-group rehearsals. In each rehearsal, TEs periodically paused the novice to explore problems of practice. For this article, we examined the TEs' pauses during rehearsal that focused on content in some way—almost half [48%] of the total number of pauses during the combined eleven rehearsals.

### 2.3 Data analysis

We reviewed the data to identify patterns and themes in how different TEs led and used rehearsals to support novices' learning of discussion facilitation (Glaser & Strauss, 1967). After initial passes of the data and the realization that TEs spent significant time on content, we refined our purpose to emphasize how TEs worked on content through rehearsals of discussion facilitation. We then reduced the data and honed-in on the videos of rehearsals as our primary data source (Miles & Huberman, 1994).

We used Studiocode software to analyze the videos of rehearsal systematically and to facilitate comparison of the different rehearsals on a consistent set of features. We worked with the larger cross-content group of researchers to construct a “code window” that specified codes we would apply to the rehearsal videos across content areas in order to facilitate subject-specific and across-subject analysis of rehearsals of discussion facilitation. This code window (or list of codes organized thematically) emerged from multiple passes of rehearsal video data across different content areas. We engaged in an iterative process of identifying patterns we noted in the video data, incorporating those patterns in the code window, testing out the code window by watching additional videos, discussing how well the code window reflected the video data, revising the code window to capture the video data more completely, and then watching more video to verify the code window or further refine it.

Once the code window was fully developed the two lead authors for this paper applied it systematically to the history/social science methods rehearsal videos from the second year of data collected. Every video of rehearsals was viewed and coded by both the first and second authors. Differences in coding were resolved through discussion and made note of in a coding guide to support future coding.

Using Studiocode's matrix feature we identified that 70% of the instances where we saw attention to content happened during *pauses* in the rehearsals. Therefore, we focused in further to examine those pauses that also included attention to content (some pauses focused on other aspects of practice; almost half of the pauses included attention to content—48%). Through multiple viewings of those pauses with content, we identified specific findings about the ways in which TEs attend to content during rehearsals of discussion facilitation. We share these findings below along with examples transcribed from the rehearsal videos that led us to identify these findings.

## 3 Findings

Our analysis revealed that all three participants used rehearsals as sites to work with novices on pedagogical issues relating to content knowledge while working on core instructional practices. In each rehearsal, novices facilitated a discussion of a guiding or central question alongside a set of sources (including written text and photographs or other images) and encouraged their peers (who pretended to be K-12 students) to develop evidence-based interpretations of historical and social issues. For example, in one secondary lesson on Inca Emperor Atahualpa's encounter with the Spanish in TE1-SEC's course, the novice fielded her peers' different interpretations of the encounter based on their reading of sources that spoke to the encounter. Or, in one first grade lesson on perspective in TE3-ELE's course, a novice teacher used details in two photographs and a score report from a football game to guide her peers' consideration of how two people can be in the same place and have different reactions to the same event. In these evidence-based interpretive discussions, we found a variety of structures for negotiating issues of content within approximations as well as a variety of aspects of content that TEs worked on with novices during rehearsals while emphasizing core instructional practices.

### 3.1 Simultaneous focus on content and core practices

In guiding novices' facilitation of discussion during rehearsals, TEs attended to content with novices when working on drawing out students' interpretive thinking about content and when helping novices hear and use the content in students' utterances. In other words, we highlight attention to content through work on two key components of the practice of facilitating discussion in our framework (see Fig. 1): engaging students as sense-makers and orienting students to each other. A focus on practice did not preclude a focus on content.

Working on content while engaging students as sense-makers took place in both secondary and elementary methods classes. In one secondary rehearsal of a lesson about the impact of imperialism on India, TE2-SEC paused the discussion when the novice's efforts at facilitating were met with total silence by her classmates. TE2-SEC advised, “That might be a point where you, either ask her to give an example or give an example yourself, so say like ‘Shauna's arguing that the Indian textbook and the British textbook are talking about different things. For example, the British are highlighting railroads, while the Indians are highlighting racial discrimination. Why would they do that?’ So, the kids have something to, like, hang on in their discussion.”

In the TE's suggestion, we see an effort to frame a discussion move that shows students that they are faced with competing interpretations and clarifies that their role is to understand why two interpretations of British imperialism in India exist. The TE phrases a question that clearly calls for students to share their thinking about a content-based problem. In an elementary example, TE3-ELE prompted a novice to rephrase a question that would engage students as sense-makers and elicit their thinking about the fairness of their school's playground equipment rather than phrasing the question for the novice: “So go back to rephrasing that question. Rewind a little and: how would you ask that question to have us get where you want?” This prompt was offered after imagining the possible ways first graders might respond to the novice's initial set of questions and a class discussion of how best to phrase a series of questions that would engage first graders as sense-makers and elicit their interpretive thinking about fairness in the context of their playground. In both examples, we see attention to a central component of a core practice (engaging students as sense-makers as part of discussion facilitation) grounded in the specific content being taught during a pause in rehearsals.

We also observed TEs working on content while orienting students to each other, another component of the core practice of discussion facilitation. Specifically, TEs encouraged novices to listen and respond to students' thinking by focusing on the content in students' utterances. In a rehearsal of a lesson on changes in a local lake over time (an integrated social studies and science lesson for 3rd grade), a novice contrasted a photograph of the lake when it was healthy and vibrant with a photograph of the same lake that had dried up several years later to prompt discussion. When the novice asked her peers what they think happened, a classmate participating in the rehearsal as a 3rd grader said she thought the lake has dried up and died. When the novice moved on to ask another, separate question, TE3-ELE interrupted:

I would pause there. That's a big statement that I think your students would potentially come up with, but I would sit with that statement for a little bit. – Who else?– cause that's big, you want to see that other people are getting that idea.

The novice then backtracked and instead of asking a new question, followed up on her peer's comment by asking, “what makes you say that?” When the peer answered, the rehearsing novice said, “good observation.” Here, again, TE3-ELE paused the rehearsal and said, “Instead of saying good observation, ask people ‘what do other people think of that idea?’” and later suggested the wording, “Does anyone want to add on to what Emily or Riley said?” as ways to prompt the novice to get students to consider each other's ideas about the interpretation that the lake has dried up and died.

Similarly, in a secondary rehearsal of a lesson on whether the Middle Ages were truly a time of stagnation, TE2-SEC suggested a way to orient students to each other by prompting students to consider each other's interpretations of the Middle Ages:

The more you can hold up to say “Zoe says this,” like “Zoe just said that they're old, that they're relying on old knowledge” or like “the textbook says this, but Brian read this last sentence, what's this sentence saying? Which side do you think is right? Were they relying on old knowledge or were they doing this deep new study?” Does that make sense?

In these rehearsal pauses, we see TEs prompting novices to consider the content in their (pretend) students' utterances and encouraging novices to provide space for the other participating students to consider their peers' ideas about the content. In these ways, participating TEs attending to content *as* they focused on components (engaging students as sense-makers, orienting students to each other) of a core K-12 instructional practice during a rehearsal. Attention to instructional practice did not preclude attention to content, and may have even enabled it.

### 3.2 The range of content worked on within rehearsals of teaching

Through pauses in rehearsals of discussion facilitation, TEs highlighted inquiry practices and concepts in history and social sciences as well as PCK for teaching these disciplines. During the pauses in rehearsals that focused on content, TEs' commentary demonstrated what orienting students to the discipline can mean by attending to the structure of the discipline, how knowledge is constructed and verified, how students might think about the content, and how to teach the content so that it is accessible to students.

In one secondary methods exchange, TE1-SEC paused a rehearsal of a lesson focused on Lexington Green and highlighted how one might orient students to the discipline by attending to both content knowledge and PCK related to disciplinary practices and concepts in history. The rehearsing novice began the lesson with a deliberation of the reliability of one source without introducing the central historical question that was the purpose of the discussion and the reason for reading the source. TE1-SEC first paused the novice to explain the importance of introducing the central question and purpose of the discussion at the beginning of the lesson before considering authorship and other indicators of reliability. The novice then re-started the discussion by explaining that their goal together during the discussion was to figure out what happened at Lexington Green. The novice facilitated discussion of what one document said happened at Lexington Green and then asked her peers if they believed what that document had to say about their central question. At that point, TE1-SEC paused again:

First of all, do you see how once you pose the central historical question then you had to circle back to do the sourcing? … It works in general better to get how the document answers the central historical question first so we're all on the same page and then go to criticizing the source … The second thing is … You keep saying ‘you're right, you're right,’ and it's a little bit of a tweak, but in the context of a discussion, you want to affirm kids, but maybe not in that right/wrong way because the whole idea is that we're putting a lot of interpretations on the table and you don't want to give them the notion that you're targeting one thing.

The TE's comments highlight several aspects of content and how one might orient students to the discipline through discussion facilitation. One way to read these comments is that the TE is focused on key lesson features and how to sequence questions so that students can get involved in the interpretive work of history while considering sources and responding to a guiding question with different perspectives. Another way to read these comments is that the TE is trying to teach novices that the purpose of history is to develop interpretations, that reliability is not a static quality but one that relies on the questions being pursued, and that there may not be a single interpretation of the topic they are considering that is right—all key concepts for understanding inquiry in history.

In this same rehearsal, we observed one additional pause that highlighted both knowledge of disciplinary practices and concepts and related PCK—allowing the class to work on orienting students to the discipline in another way. When the rehearsing novice trudged forward in the discussion and directed her peers to look at a second document, TE1-SEC paused to say,

What might be another way to transition to another document that could get kids to clamor for it … how could you get kids to say, ‘we would need another document?’ … Are we done? do we have the final word here? … Or would you want some other information? … Historians always want more information, we're never happy with just one account. Well, whose perspective would you want … ?

Here, the TE emphasizes the importance of corroboration and grounding historical interpretation in multiple sources and through attention to different perspectives while also presenting a way to make these concepts accessible to students. These exchanges during the Lexington Green rehearsal highlight key features of content knowledge (particularly knowledge of disciplinary practices and concepts) and PCK through a novice's discussion facilitation.

In another rehearsal, TE1-SEC paused to work on content knowledge about a specific topic with novices in the context of interpreting Lincoln's motivation in issuing the Emancipation Proclamation. In this example, the TE demonstrated that attending to accuracy of knowledge is yet another way of orienting students to the discipline. These central questions framed the discussion: Why did Lincoln free the slaves? Was it moral conviction or military necessity? During the novice's discussion facilitation, her peers struggled to come up with plausible arguments about Lincoln's motivations. TE1-SEC diagnosed this as a problem with participants' knowledge of the Civil War era and paused the rehearsal:

What I'm sensing is a lot of confusion around the timeline and this particular paragraph. And so, at this point I would recommend that you stabilize the content … pause and let's get some things on the table. … it's after Antietam. The fear is not that the Confederates are going to win. The fear is that the Union is going to win, but will slaves be free? … The issue is—there's all this pressure, we're winning, let's end the war— but if he hadn't done the Emancipation Proclamation the slaves would have remained (the ones who hadn't escaped) would have remained enslaved. The Union would have been connected, but the slaves would have remained enslaved. Therefore, he was moved to issue the Emancipation Proclamation before that happened, which is Frederick Douglass's argument for why [the issuing of the Emancipation Proclamation] was [about] moral conviction.

In her commentary, the TE teaches the novices about the trajectory of the Civil War and Lincoln's concerns given the context of events. In re-setting novices' content knowledge about this time period the TE opens the path for them to develop plausible interpretations about the Emancipation Proclamation while also conveying the role of a teacher is also to monitor the accuracy of information so that students are positioned to do the interpretive work of history. The TE's comments also convey that the purpose of discussion is not simply to get students to talk but to understand the content—in this case, to understand the meaning of the sources under consideration.^1^ To understand sources means understanding the context of authors' lives and their views of the world as well as comparing sources to grasp their full meaning. This complex interpretive work with sources is made possible with accurate knowledge of the Civil War era, and the rehearsal of this discussion provides space to attend to this important topical knowledge. In this and other examples, the TE's work on content during pauses in the rehearsal highlight how one might orient students to the discipline while facilitating a discussion.

### 3.3 Approaches to working on content during rehearsals

Looking across all of the content-focused pauses, we noticed that TEs used four different approaches to structure pauses during rehearsal across elementary and secondary contexts: TEs talked while novices listened, gave specific directions or recommendations for the rehearsing novice to follow, had one-on-one interactions with the rehearsing novice, or facilitated a whole-class conversation about aspects of leading a discussion.

The rehearsal of the first-grade discussion about perspectives on the outcome of a recent football game reveal several of these approaches. In this example, the rehearsing novice teacher brought together two photographs of people reacting to a football game wearing clothes that show their allegiance to one team or the other as well as a score report with symbols for each team that students might use to explain the different reactions in the photographs. Although a football game is not necessarily social studies content, the focus on teaching perspective and the related concept that people can have different perspectives about the same event is a central concept in the social studies. In this rehearsal, TE3-ELE spoke while the rehearsing novice listened, she had a one-on-one exchange with the rehearsing novice, and she facilitated a whole-class conversation about discussion facilitation during pauses in the rehearsal. Throughout these particular pauses, the TE highlights how one might orient students to the text to support understanding of content while facilitating discussion.

Here, TE3-ELE paused the rehearsal to speak about key questions that could orient students to the visual texts while the novice (and others) listened:

In terms of observation questions that I think are important for this is “what's happening with her face? What do you notice about her face?” and then the inference question is “how do you think she feels?” That'll be an important contrast I think with your next [photograph].

The TE spoke here after the novice listed a number of questions she could ask about the picture without a clear sense of purpose. The TE's direct comment tried to focus the rehearsing novice on the aspects of the sources that could help achieve the purpose of the discussion and the kinds of questions that might help students work with the sources effectively.

In other moments during the same rehearsal, TE3-ELE paused to have one-on-one exchanges with the novice teacher as well as to facilitate a group conversation with all of the novice teachers in the room. This one-on-one exchange focused on affirming the novice's thinking about students and her facilitation moves in orienting students to the text thus far as well as making visible key discussion moves the novice had made:

Rehearsing Teacher: I think [my first graders are] gonna know.

TE3-ELE: They're gonna know. It's hard to live anywhere near us and not know [the symbols for this football team]. So, you're asking some observation and inference questions, it sounds like.

RT: Yeah

TE3-ELE: And you had a nice follow up question of “how do you know?” That helps them focus on what are the details there, they are using.

At other points in this rehearsal, TE3-ELE coordinated a whole-class conversation in which all novice teachers processed the practice of discussion facilitation and how to support first-graders' work and thinking with the visual texts.

TE3-ELE and Non-Rehearsing Teachers: I think they're gonna know [how to make sense of a football game score].

RT: They will, they totally will. I still need to mask this [she points to what she's showing on the projector] ‘cause I think I want this [score report] simplified. I want to get rid of all that and get rid of that so it's strictly that.

TE3-ELE and NRTs: That's great, that's great.

TE3-ELE: And I'm not even sure – I think this is a nice thing to have in your back pocket, but I'm wondering if you'll even need it. You could just ask “why are these people happy and this person is not happy?” …

RT: I mean the other thing that I could do … if I simplify this graphic and even get rid of these, I could ask them to match the [school's] symbol to each score … like the understanding that the person with the bigger number is the winner, they could make those connections.

NRT: Yeah, they should be able to.

In this exchange among all novice teachers, the group thinks together about how first-grade students will make sense of the visual texts and develop background knowledge so that they are prepared to understand the central goal that two people can have a different perspective of the same event. Within the rehearsal space, the whole group thinks together about orienting students to the text and preparing texts so that students can achieve the desired content understandings. The TE is one of several participants in this pause and we see how rehearsal is a space for an entire class to develop a better understanding of facilitating discussion about social studies content.

In other rehearsals, we saw these same approaches in addition to one more—giving specific directions for the novice to follow or offering lines for the novice to use when facilitating discussion. For example, in the discussion about the Middle Ages, the rehearsing novice asked participants to comment on the two categories of ideas that her peers had raised without clarifying those ideas. At that moment, TE2-SEC interjected, “Name what the categories are.” The novice took up the suggestion by naming the two sets of ideas up for consideration as shared by her peers, and proceeded with the discussion. A few minutes later, when one of the rehearsing novices' peers referenced the context of the sources they discussed, TE2-SEC made a specific recommendation to highlight the disciplinary thinking that had just occurred: “Can you name the way he's contextualizing? So, you might name and describe what he's doing.” And in one other example from the discussion about British imperialism in India, TE2-SEC stopped the discussion to give specific direction to the rehearsing novice: “You might just, before you go on, Vivian voiced a negative opinion about laws, but you might say ‘can anyone think of an argument you can make for why the same law for everybody could be a good thing?’” The rehearsing novice took up the suggestion, literally repeating the TE's line, as she proceeded with the discussion. This kind of guidance offered novices language and direction for facilitating discussion of the content during rehearsal while minimizing the length of time and deliberation in pauses.

## 4 Discussion

Our goal in this investigation was to understand how, if at all, history/social science TEs use rehearsals of discussion facilitation as a space for engaging with novices around the particularities of history/social science content. We found that rehearsals provided rich spaces for working on content. We believe our findings contribute to and extend the growing body of literature on rehearsals in practice-based teacher education (e.g., Kavanagh et al., 2019; Lampert et al., 2013a, 2013b; Schutz, Danielson, & Cohen, 2019) by offering examples in which TEs entwine work on practice with content. Moreover, that TEs in this study conceptualized content knowledge to include interpretive disciplinary practices *so that* such ways of thinking might be worked on in whole-class discussions, suggests the value of using core instructional practices to engage students in high-level subject matter learning (Grossman et al, 2009).

Rehearsals, we found, offered TEs opportunities not only to attend to particular components of practice, such as engaging students as sense-makers, but also to attend to variations in novice teachers' elicitations, student responses, and disciplinary goals. While novice teachers are commonly granted opportunities to learn about questions that prompt student thinking about content and plan for discussions, rehearsals offered something more: opportunities to practice making judgments about how best to engage students as sense-makers, orient students to each other, orient students to text, and orient students to the discipline in response to very real input from discussion participants. This finding is significant to contemporary debates about practice-based teacher education in which some scholars are expressing fears about what Zeichner (2012) has called “the turn to practice in teacher education.” Zeichner (2012), Kennedy (2016), and Philip et al. (2018) have all expressed a wariness that focusing on practice means decontextualizing the visible behaviors of teaching from their purposes and contexts. While any instance of teaching has many contexts (e.g. sociopolitical context, sociocultural context, local community context, etc.), one of these contexts is disciplinary. Each classroom discussion exists within the broader context of the particular content being discussed and that content's location in a larger disciplinary tradition of meaning making. Therefore, the moves a teacher makes when facilitating a discussion cannot be decontextualized from those moves' disciplinary context without effects on what and how students learn. Our findings indicate that it is possible to work on a practice *without* decontextualizing visible teaching behaviors from their disciplinary context and disciplinary purposes. While our investigation does not center on other contexts of teaching (e.g. sociopolitical, sociocultural, etc.), future research could use a similar methodology to study these contexts.

The framework we developed for focusing on the different components of the act of facilitating a discussion with children was focused on history discussions only but these rehearsals covered social studies more broadly, especially in the elementary classrooms. Based on our analyses of rehearsals, we felt the framework applied to the range of discussions we examined and therefore would be comfortable shifting the name of the framework to specify it as a framework for facilitating social studies discussions, especially with the broader conception of orienting students to content that is evidence of the work done during rehearsals.

This study does not clarify whether any particular approach to structuring rehearsal is more effective in supporting novices' capacity for discussion facilitation, but this could be a future investigation. What do novice teachers learn when fed a line versus when participating in a conversation about an aspect of practice or watching an interaction? In addition, we have yet to explore the ways in which rehearsals enable other aspects of novice teachers' learning about discussion facilitation. Last, we could explore how rehearsals—and discussion facilitation—vary when the TEs focus on one type of discussion as opposed to another (e.g., Parker & Hess, 2001). Regardless, at this point we can say that the TEs we studied took different approaches to pauses during rehearsals, elaborating our sense of rehearsal as a TE pedagogy and highlighting the use of pauses within a rehearsal as a space for working on content with novice teachers as they learn to facilitate discussion.

At a broader level, our findings speak to contemporary conversations about rehearsals and practice-based teacher education more globally. Some scholars argue that focusing teacher learning on practice leads to a technocratic approach to teaching that privileges standardization over improvisation and rote behaviors over thought (Philip, 2019; Philip et al., 2018). However, fine-grained analyses of rehearsals continue to illustrate that expert TEs can use the details of practice to support novices in investigating and embodying the complex relationships between how disciplines are structured, how knowledge is constructed, and how students make sense. Sometimes getting closer to something (a painting, a raindrop, or a teaching practice) reveals, rather than reduces, its complexity.

## Footnotes

1 The TE literally says, “You're not just getting kids to gab, you're trying to get at the deep stuff that is in these documents.”
